# Shifting Rice Cropping Systems Mitigates Ecological Footprints and Enhances Grain Yield in Central China

**DOI:** 10.3389/fpls.2022.895402

**Published:** 2022-05-04

**Authors:** Yong Zhou, Ke Liu, Matthew Tom Harrison, Shah Fahad, Songling Gong, Bo Zhu, Zhangyong Liu

**Affiliations:** ^1^Hubei Collaborative Innovation Center for Grain Industry, College of Agriculture, Yangtze University, Jingzhou, China; ^2^Hubei Province Research Center of Engineering Technology for Utilization of Botanical Functional Ingredients, College of Life Science and Technology, Hubei Engineering University, Xiaogan, China; ^3^Tasmanian Institute of Agriculture, University of Tasmania, Burnie, TAS, Australia; ^4^Hainan Key Laboratory for Sustainable Utilization of Tropical Bioresource, College of Tropical Crops, Hainan University, Haikou, China; ^5^Department of Agronomy, The University of Haripur, Haripur, Pakistan

**Keywords:** double rice, conversion, ratoon rice, rice-wheat, carbon footprint, nitrogen footprint, water footprint

## Abstract

Intensive cereal production has brought about increasingly serious environmental threats, including global warming, environmental acidification, and water shortage. As an important grain producer in the world, the rice cultivation system in central China has undergone excessive changes in the past few decades. However, few articles focused on the environmental impacts of these shifts from the perspective of ecological footprints. In this study, a 2-year field trial was carried out in Hubei province, China, to gain insight into carbon footprint (CF), nitrogen footprint (NF), and water footprint (WF) performance. The three treatments were, namely, double-rice system (DR), ratoon rice system (RR), and rice-wheat system (RW). Results demonstrated that RR significantly increased the grain yield by 10.22–15.09% compared with DR, while there was no significant difference in the grain yield between RW and DR in 2018–2019. All of the calculation results by three footprint approaches followed the order: RR < RW < DR; meanwhile, RR was always significantly lower than DR. Methane and NH_3_ field emissions were the hotspots of CF and NF, respectively. Blue WF accounts for 40.90–42.71% of DR, which was significantly higher than that of RR and RW, primarily because DR needs a lot of irrigation water in both seasons. The gray WF of RW was higher than those of DR and RR, mainly due to the higher application rate of N fertilizer. In conclusion, RR possesses the characteristics of low agricultural inputs and high grain yield and can reduce CF, NF, and WF, considering the future conditions of rural societal developments and rapid demographic changes; we highlighted that the RR could be a cleaner and sustainable approach to grain production.

## Introduction

Agriculture production is facing a daunting influence of climate change to meet the increasing food consumption demands (Ahmed et al., 2019; Liu et al., 2021; Yan et al., 2022). In 2020, close to 12% of the global population was food insecure (FAO et al., 2021). For all that, FAO et al. (2018) reported that climate variability and extreme climate were negatively affecting agricultural productivity at the global, national, and local levels, which was reflected in the change of crop yields, cropping area, and intensity. Cereal is the main source of human food, and cereal production of China accounted for approximately 23.13% of the world’s cereal production (FAOSTAT, 2020). Rice (*Oryza sativa* L.) is one of the most important cereals in China, with the cropping area and yield accounting for 25.44 and 33.64%, respectively (Liu et al., 2020a; National Bureau of Statistics [NBS], 2022). Currently, rice production is facing three major environmental problems, namely, (1) paddy rice production is a primary source of greenhouse gas (GHG); the methane emissions from paddy fields reach 33–40 Tg year^–1^ from 2000 to 2009, accounting for approximately 18% of global anthropogenic methane emissions (Ciais et al., 2013); (2) farmers usually overuse synthetic chemicals (especially, nitrogen fertilizer) to achieve high yields, which have led China to become the world’s largest consumer of nitrogen fertilizer with a proportion of 37.6% (Ray et al., 2012). However, there is an extremely low nitrogen use efficiency (NUE) at 35% in rice production in China (IPCC, 2013). Therefore, large amounts of reactive nitrogen (Nr) leak into the environment due to the above issue, resulting in serious environmental problems such as global warming, eutrophication, and environment acidification (Tallentire et al., 2018); (3) rice production consumes a lot of water. The global available water shortage or regional extreme imbalance caused by climate change poses a serious challenge to the allocation of water resources in rice production (FAO, 2017).

In recent decades, the rice cropping system in central China has undergone great changes, i.e., cultivation area of traditional double-rice system (DR) has decreased, while ratoon rice system (RR) and rice-wheat system (RW) have increased rapidly, due to lower economic profit and labor shortage (Chen et al., 2020; Jiang et al., 2020; Liu et al., 2020b). The conversion of the rice cropping system not only significantly affects field GHG emissions but also changes resource consumption and indirect environmental emissions of agricultural inputs (IPCC, 2014; Liu et al., 2020c). Therefore, it is highly necessary to develop effective methods to evaluate the GHG emissions, Nr losses, and water consumption for this conversion. In recent years, footprint indicators, such as carbon footprint (CF), nitrogen footprint (NF), and water footprint (WF), were widely used by ecologists to estimate the environmental impact of agricultural system (Pierer et al., 2014; Shrestha et al., 2017; Kashyap and Agarwal, 2021; Zhang and Xu, 2021). The calculation of CF and NF is usually based on the “cradle to grave” theory of life cycle assessment (LCA) (British Standards Institution [BSI], 2008). The CF in the agricultural domain refers to all direct (i.e., field) and indirect (i.e., agricultural inputs) GHG emissions from agricultural production, which is expressed as the CO_2_ equivalent (Jiang et al., 2019). Similarly, the NF refers to the Nr losses accompanied within the whole life cycle process of crop cultivation quantified in kg N equivalent, including NH_3_ volatilization, N_2_O emissions, NO_3_^–^, and NH_4_^+^ leaching (Chen et al., 2020). In this study, WF is based on the concept of virtual water, which is divided into three parts: blue, green, and gray water. Blue water refers to irrigation water, green water mainly refers to natural precipitation, and gray water refers to the freshwater consumption required to dilute nitrogen pollution (Hoekstra et al., 2011; Mekonnen and Hoekstra, 2011).

Many previous studies have reported the CF or NF of DR separately (Sun et al., 2019; Jiang et al., 2020; Zhang and Xu, 2021), Chen et al. (2020) proposed that CF of DR in China was lower than RW, while NF was higher than RW. Xu et al. (2022) suggested that the conversion of DR to RR in central China could reduce CF. Jiang et al. (2020) reported that the conversion of DR to maize rice in east China could also reduce CF. Xu et al. (2020b) compared the WF and NF of different cultivation modes in North China Plain. However, to our knowledge, few studies have applied the CF, NF, and WF interconnect method to comprehensively evaluate DR, RR, and RW. Therefore, the objectives of this study were to (1) evaluate CF, NF, and WF of the three rice-cropping systems and identify their contribution to hotspots and (2) identify the rice production system with higher yield and less environmental impacts and provide recommendations for future research and policymaking.

## Materials and Methods

### Site Description

The field experiment was conducted in the trial bases of Sanhu farm, Jiangling county, Jingzhou city, Hubei province, China (30°12′N, 112°31′E), between March 2017 and May 2019. This area experiences a northern, humid, subtropical monsoon climate with a mean annual temperature and average annual precipitation of 16.0°–16.4° and 900–1,100 mm, respectively. The soil in this region was classified as inceptisol, while the soil properties (0–20 cm depth) were as follows: total carbon 26.44 g/kg, total nitrogen 2.44 g/kg, organic matter 28.59 g/kg, alkali hydrolyzed nitrogen 170.88 mg/kg, total phosphorus 0.38 g/kg, available phosphorus 12.67 mg/kg, total potassium 17.76 g/kg, available potassium 159 mg/kg, and pH 6.92.

### Experimental Design and Management

**FIGURE 1:**
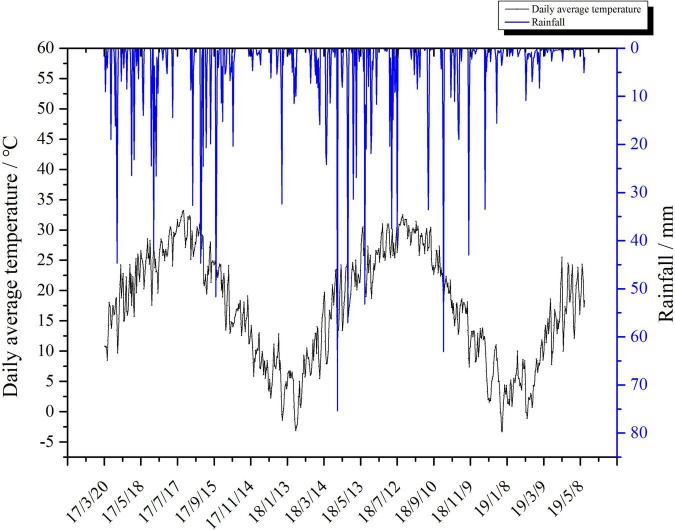
Local daily average temperature and rainfall during the test period.

Three rice cropping systems, namely, DR, RR, and RW, were subjected with a randomized block design with three replications. The size of each experimental plot was 98 m^2^ (14 m × 7 m), and a ridge with plastic film attached around each plot was established to prevent the flow of water and the fertilizer. The previous cultivation pattern was rice monoculture and residue return fields. Field management measures were according to local agronomic practices. Detailed information of agricultural inputs, including crop species, seed rate, agrochemical rate, diesel, and electricity usage are shown in Table 1; the date of sowing, transplanting, and harvest for the three cropping systems are shown in Table 2; local daily average temperature and rainfall during the test period are shown in Figure 1. Field management practices are provided as follows:

**TABLE 1:** Life cycle inventory for the three rice cropping systems.

Items	Unit	DR			RR			RW		
		Early rice	Late rice	Subtotal	First rice	Ratoon rice	Subtotal	Rice	Wheat	Subtotal
N fertilizer	kg/ha	180	180	360	200	150	350	225	90	315
P fertilizer	kg/ha	75	75	150	75	0	75	75	0	75
K fertilizer	kg/ha	180	150	330	180	0	180	180	0	180
Compound fertilizer	kg/ha	0	0	0	0	0	0	0	600	600
Diesel	L/ha	75.00	75.00	150	75.00	33.33	108.33	83.33	66.67	150.00
Electricity	kWh/ha	369.25	910.85	1,280.1	381.42	82.23	463.65	375	0	375
Herbicides	kg/ha	1.31	1.31	2.62	1.31	0	1.31	1.31	0	1.31
Insecticides	kg/ha	0.8	0.8	1.6	0.8	0	0.8	0.8	0	0.8
Fungicides	kg/ha	0.23	0.23	0.46	0.23	0	0.23	0.23	0.2	0.43
Labor	person⋅d/ha	15	15	30	17	3	20	15	2	17
Rice seed	kg/ha	18.75	18.75	37.5	18.75	0	18.75	18.75	0	18.75
Wheat seed	kg/ha	0	0	0	0	0	0	0	225	225

*DR, double-rice system; RR, ratoon rice system; RW, rice-wheat system.*

**TABLE 2:** Date of sowing, transplanting, and harvest for the three cropping systems.

Year	Treatment	Sowing – transplanting – harvest (mm/dd)	
		1st season	2nd season
2017–2018			
	DR	03/29-05/02-07/20	06/23-07/27-11/03
	RR	03/29-05/02-08/15	08/16-11/03
	RW	05/12-06/06-09/25	11/09-05/16
2018-2019			
	DR	03/25-05/03-07/18	06/22-07/27-11/01
	RR	03/25-05/03-08/10	08/11-10/23
	RW	05/09-06/04-09/19	11/01-05/10

*DR, double-rice system; RR, ratoon rice system; RW, rice-wheat system.*

#### Double-Rice System

The early rice and late rice cultivar were Liangyou 287 and Jinyou 207, respectively. Transplanting density of both early and late rice was 26.70 cm × 16.70 cm, with three seedlings in each hole. Both the late and early rice received the same rate of N and P fertilizer, with 180 kg N ha^–1^, 75 kg P_2_O_5_ ha^–1^, 180 and 150 kg K_2_O ha^–1^ were applied to early and late rice, respectively. P fertilizer was applied once a season, while the rate of basal fertilizer and top-dressing at the panicle of K fertilizer was 5:5. Usage of N fertilizer in early rice was divided into basal fertilizer (50%), top-dressing at tillering (20%), and top-dressing at panicle (30%), while the ratio of late rice was 2:2:1.

#### Ratoon Rice System

The ratoon rice cultivar was Liangyou 6326. Transplanting density of first-season rice was the same as that of early rice. The fertilizer rate of first-season rice was 200 kg N ha^–1^, 75 kg P_2_O_5_ ha^–1^, and 180 kg K_2_O ha^–1^. Only 150 kg N fertilizer was applied in the ratoon season (sprout-promoting fertilizer:seedling-raising fertilizer = 1:1). Sprout-promoting fertilizer was applied 10 days after full heading in the first season, and seedling-raising fertilizer was applied 10 days after harvest in the first season. The stubble height after harvest in the first season was about 40 cm.

#### Rice-Wheat System

The rice and wheat varieties were Longliangyouhuazhan and Zhengmai 9023, respectively. During rice cultivation, the transplanting density was 26.70 cm × 16.70 cm, the proportion of N fertilizer was base fertilizer:tillering fertilizer:earing fertilizer = 4:3:3; P fertilizer was applied as base fertilizer at one time, and the proportion of K fertilizer was base fertilizer:ear fertilizer = 1:1; water managements were irrigation in the early stage, drying in the middle stage, and alternating dry and wet in the later stage. After the harvest of rice, wheat was seeded by a shallow rotary cultivator with a sowing amount of 225 kg ha^–1^ and a basic seedling density of 3 × 106 plants ha^–1^. During the growth period of wheat, compound fertilizer (N:P_2_O_5_:K_2_O = 16:10:22), 600 kg ha^–1^ were applied as basal fertilizer, while top application nitrogen of 90 kg ha^–1^ was applied. Other field managements were the same as the conventional paddies.

### System Boundary and Functional Unit

According to PAS 2050:2011 (British Standards Institution [BSI], and Carbon Trust, 2011), we set the system boundary from agricultural inputs production to the crops harvest (from cradle to gate) for CF and NF. CF calculation did not involve changes in soil carbon content based on the PAS 2050 principle. The functional unit of CF, NF, and WF was 1 ton grain yield.

### Calculation of Carbon Footprint

In accordance with the definition of IPCC 2006, the CF refers to all indirect and direct greenhouse gas (GHG) emissions within the whole life cycle process of crop production quantified as CO_2_ equivalents (CO_2_-eq). The indirect GHG emissions include manufacturing, transportation, storage, and application of agricultural inputs, while the direct emissions refer to CO_2_, N_2_O, and CH_4_ emissions from soils; direct CO_2_ emission was not considered in this study because of the higher CO_2_ fixation by crops than their emissions (IPCC, 2013). Therefore, the CF was calculated according to the following equations (Pandey and Agrawal, 2014; Wang et al., 2016):


(1) $$C\,F=\frac{\sum \left(A\,I\,i\times E\,F\,i\right)+E\,\left(N_{2}\,O\right)\times 298+C\,E\,\left(C\,H_{4}\right)\times 25}{Y}$$

where, AI_*i*_ is the amount of agricultural inputs including fertilizers, pesticides, electricity, diesel, seeds, and labor. EF_*i*_ is the carbon emission factors of agricultural inputs as shown in Table 3, which is quoted from Ecoinvent version 2.2 (Swiss Centre for Life Cycle Inventories, Switzerland) and Chinese Life Cycle Database (CLCD version 0.8). *CE (CH_4_)* and *E(N_2_O)* are the amount of methane and nitrous oxide measured directly from soils. The global warming potential (GWP) coefficients of N_2_O and CH_4_ at a 100-year time horizon (IPCC, 2013) are 298 and 25, expressed in kg CO_2_-eq kg^–1^, respectively. Y is grain yield per unit area, t ha^–1^.

**TABLE 3:** Emission factors of carbon and nitrogen footprints.

Agriculture inputs	Carbon footprint (CF)		Nitrogen footprint (NF)	
	Value	Unit	Value	Unit
N fertilizer	1.53	kg CO_2_-eq kg^–1^	8.90E−04	kg N-eq kg^–1^
P fertilizer	1.63	kg CO_2_-eq kg^–1^	5.40E−04	kg N-eq kg^–1^
K fertilizer	0.65	kg CO_2_-eq kg^–1^	3.00E−05	kg N-eq kg^–1^
Compound fertilizer	1.77	kg CO_2_-eq kg^–1^	2.30E−04	kg N-eq kg^–1^
Diesel	4.99	kg CO_2_-eq L^–1^	5.36E−03	kg N-eq L^–1^
Electricity	0.82	kg CO_2_-eq kWh^–1^	1.20E−04	kg N-eq kWh^–1^
Herbicides	16.61	kg CO_2_-eq kg^–1^	3.53E−03	kg N-eq kg^–1^
Insecticides	10.15	kg CO_2_-eq kg^–1^	4.49E−03	kg N-eq kg^–1^
Fungicides	10.5	kg CO_2_-eq kg^–1^	7.05E−03	kg N-eq kg^–1^
Labor	0.86	kg CO_2_-eq person^–1^ d^–1^	0	kg N-eq person^–1^ d^–1^
Rice seed	1.84	kg CO_2_-eq kg^–1^	7.60E−04	kg N-eq kg^–1^
Wheat seed	0.58	kg CO_2_-eq kg^–1^	2.40E−04	kg N-eq kg^–1^

*1. GHG emission factors of pesticides and seeds were quoted from Ecoinvent version 2.2 (Swiss Centre for Life Cycle Inventories, Switzerland), other factors were quoted from Chinese Life Cycle Database (CLCD version 0.7, IKE Environmental Technology CO., Ltd, China); 2. active nitrogen emission factors were from eBalance version 3.0 (IKE Environment Technology Co., Ltd, China); and 3. diesel includes two parts: production and consumption.*

### Direct Greenhouse Gas Emissions

A static chamber (100 cm × 50 cm × 50 cm) method and gas chromatography were employed to measure direct GHG emissions (i.e., N_2_O and CH_4_) from soils. The material of the chamber was Plexiglas, and a tinfoil was wrapped around the chamber to keep the internal temperature from changing drastically. N_2_O and CH_4_ fluxes were collected between 9:00 and 11:00 one time per week; gas samples were collected at 0, 5, and 10 min after the chamber was closed. Concentrations of N_2_O and CH_4_ were analyzed by a gas chromatograph (Agilent 7890A), in which, N_2_O was detected with an electron capture detector (ECD), and CH_4_ was detected with a flame ionization detector (FCD). The N_2_O and CH_4_ fluxes were calculated using a linear increase of gas concentration over time. The calculation formula is as follows:


(2) $$F=\frac{d\,c}{d\,t}\cdot h\cdot \rho \cdot \frac{273}{273+T}$$

where F is N_2_O and CH_4_ emission rates, the units of N_2_O and CH_4_ are μg (m^2^ h)^–1^ and mg (m^2^ h)^–1^, respectively; dc/dt is the rate of change of gas concentration in the chamber with time during the sampling process; the units of N_2_O and CH_4_ are μl (m^3^ h)^–1^ and ml (m^3^ h)^–1^, respectively; h is the height of the chamber, 1.0 m; ρ is the gas density at standard atmospheric pressure; the unit of N_2_O is 1.964 kg m^–3^, and the unit of CH_4_ is 0.714 kg m^–3^; 273 is the absolute temperature (K); T is the temperature inside the chamber during sampling (°).

### Calculation of Nitrogen Footprint

Nitrogen footprint refers to the environmental impacts of Nr loss on water, air, and soil based on ISO 14044 (ISO, 2006) and CML2002 (Guinée et al., 2002) methodology, which was characterized as eutrophication potential (EP) in this research. Similar to the CF, the calculation of NF includes indirect emissions of agricultural inputs and direct emissions from soils. On-field Nr loss mainly includes NH_3_ volatilization, N_2_O emission, and NO_3_^–^ and NH_4_^+^ leaching, respectively. The NF was calculated as follows (Chen et al., 2020):


(3) $$N\,F=\frac{\sum \left(A\,I\,j\times \text{E}\,F\,j\right)+N\,V\,\left(N\,H_{3}\right)+N\,L\,\left({\text{NO}}_{3}^{-}\right)+N\,L\,\left(N\,H_{4}^{+}\right)+N\,E\,\left(N_{2}\,O\right)}{Y}$$

where AI_*j*_ is the amount of agricultural inputs, EF_*j*_ is the nitrogen emission factors of agricultural inputs (Table 3). *NV(NH_3_)*, *NE(N_2_O)*, *NL(NO_3_^–^)*, and *NL(NH_4_^+^)* are the amount of NH_3_ volatilization, N_2_O emissions, and NO_3_^–^ and NH_4_^+^ leaching from the field, expressed in kg N-eq kg^–1^, respectively.


(4) NV(NH)3=N×α×1714× 0.833

(5) $$\mathrm{NL}\,\left({\mathrm{NO}}_{3}^{-}\right)=N\times \beta \times \frac{62}{14}\times 0.238$$

(6) $$\mathrm{NL}\,\left({\mathrm{NH}}_{4}^{+}\right)=N\times \gamma \times \frac{18}{14}\times 0.786$$

(7) NE⁢(N2⁢O)=E⁢(N⁢O2)×4428× 0.476

where N is the nitrogen fertilizer rate (kg ha^–1^). α, β, and γ are the coefficients of NH_3_ volatilization loss, NO_3_^–^, and NH_4_^+^ leaching, respectively. The α value is 0.142 (Xia et al., 2021). In accordance with the manual for China fertilizer leaching coefficients, the β value is 0.066 for DR and RR, 0.060 for RW; the γ values are 0.339, 0.165, and 0.19% for DR, RR, and RW, respectively. 17/14, 62/14, 18/14, and 44/28 are the molecular weight ratios of NH_3_ to NH_3_-N, NO_3_^–^ to NO_3_^–^-N, NH_4_^+^ to NH_4_^+^-N, and N_2_O to N_2_O-N, respectively. 0.833, 0.238, 0.786, and 0.476 are the EP coefficients of NH_3_, NO_3_^–^, NH_4_^+^, and N_2_O at a 100-year time horizon, which are sourced from the CML2002 (Guinée et al., 2002).

### Calculation of Water Footprint

In this article, the utilization of blue water and green water resources is based on the measured results of the local 2-year experiment. The pollution caused by N fertilizer application was mainly considered when calculating gray WF, and the critical dilution volume method was adopted to calculate gray water demand. The calculation formulas are as follows:


(8) $$\text{WF}={\text{WF}}_{b\,l\,u\,e}+{\mathrm{WF}}_{g\,r\,e\,e\,n}+{\mathrm{WF}}_{g\,r\,a\,y}$$

(9) $${\text{WF}}_{b\,l\,u\,e}=\frac{{\text{CWU}}_{b\,l\,u\,e}}{\text{Y}}$$

(10) $${\text{WF}}_{g\,r\,e\,e\,n}=\frac{{\text{CWU}}_{g\,r\,e\,e\,n}}{\text{Y}}$$

(11) $${\text{WF}}_{g\,r\,a\,y}=\frac{{\text{CWU}}_{g\,r\,a\,y}}{\text{Y}}$$

(12) CWUg⁢r⁢a⁢y=LpC-maxCnat

where, *WF*_*blue*_, *WF*_*green*_, and *WF*_*gray*_ refer to blue, green, and gray WF, respectively, expressed in m^3^ t^–1^, respectively. *CWU*_*blue*_, *CWU*_*green*_, and *CWU*_*gray*_ are the water consumption of blue water, green water, and gray water during crop growth period, respectively, expressed in m^3^ ha^–1^, respectively. *L*_*p*_ is the amount of pollutants entering water, and 10% of the total nitrogen application amount is usually selected (Hoekstra et al., 2011). *C*_*max*_ is the maximum acceptable pollutant concentration in water, no more than 10 mg of nitrogen per liter of drinking water (EPA, 2005); *C*_*nat*_ is the concentration of the pollutant in the water in the natural state, which is usually 0.

### Statistical Analysis

All data were presented as the means ± SE (standard error). Statistical analysis was conducted using SPSS 26.0 (SPSS Inc., IL, Chicago, United States) and Origin Pro 9.0 (OriginLab Corporation, Northampton, MA, United States). Two-way ANOVA was employed to analyze effects of years and cropping systems on the grain yield, CF, NF, and WF, followed by Duncan multiple comparison with a significance level of 5%.

## Results

### Variation of Cultivation Area and Yield Performance

**FIGURE 2:**
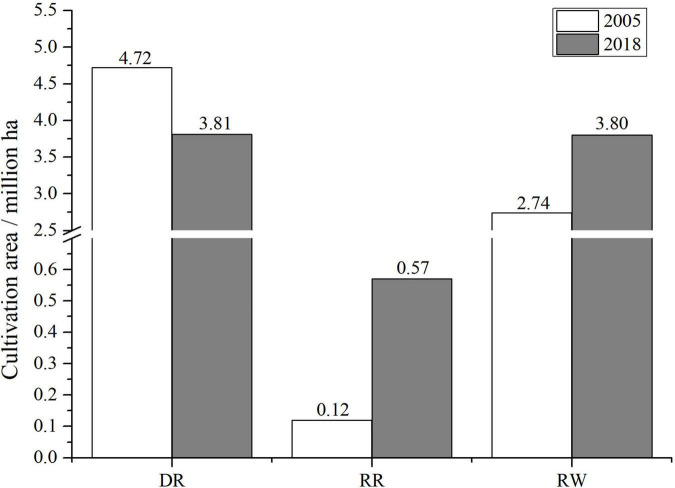
Shift of cultivation area for the three rice cropping systems in central China. DR, double-rice system; RR, ratoon rice system; RW, rice-wheat system; the original data came from Agricultural Technology Extension Station of Hubei, China.

As shown in Figure 2, cultivation area of the traditional DR in central China decreased from 4.72 million ha in 2005 to 3.81 million ha in 2018. On the contrary, the planting area of RW increased by more than 1 million ha, from 2.74 to 3.80 million ha in the same period and area. What is more amazing was that the RR increased nearly four times, from 0.12 to 0.57 million ha.

The 2-year experiment showed that there were some significant differences in grain yield among the three rice cropping systems (Table 4). In 2017–2018, the grain yield in RR and RW was significantly higher than that in DR (*P* < 0.05); however, no significant difference was detected between RR and RW. When it came to 2018–2019, the grain yield of RR was 16.83 t ha^–1^, which was significantly higher than RW (15.55 t ha^–1^) and DR (15.06 t ha^–1^); no significant difference was found between RW and DR. Additionally, the order of grain yield was RR > RW > DR in both years.

**TABLE 4:** Grain yield, carbon, nitrogen, and water footprints of the three cropping systems.

Year	Treatment	Grain yield (t/ha)	Carbon footprint (kg CO_2_-eq/t)	Nitrogen footprint (kg N-eq/t)	Water footprint (m^3^/t)
2017–2018					
	DR	12.99 ± 0.65b	1,094.13 ± 100.63a	6.59 ± 0.49a	1,338.33 ± 65.36a
	RR	14.95 ± 0.28a	567.22 ± 79.65b	5.41 ± 0.21b	1,074.81 ± 20.57c
	RW	14.72 ± 0.52a	604.99 ± 27.28b	6.23 ± 0.27a	1,183.72 ± 41.54b
2018–2019					
	DR	15.06 ± 0.24b	718.97 ± 37.65a	6.07 ± 0.26a	1,103.79 ± 17.43a
	RR	16.83 ± 0.23a	483.98 ± 14.28c	5.03 ± 0.06b	909.21 ± 12.46c
	RW	15.55 ± 0.34b	578.57 ± 55.67b	5.94 ± 0.25a	1,007.46 ± 22.20b
*F*-value					
Year (Y)		68.38**	32.15**	8.55*	135.21**
Cropping systems (C)		31.38**	68.00**	25.03**	64.25**
Y × C		4.01*	14.37**	0.24*^ns^*	1.68*^ns^*

DR, double-rice system; RR, ratoon rice system; RW, rice-wheat system. Mean ± standard deviation; different lower-case letters in the same year and column indicate the significantly differences (P < 0.05); * significant at P < 0.05, ** significant at P < 0.01

*^ns^P > 0.05.*

### Carbon Emissions and Carbon Footprint

**FIGURE 3:**
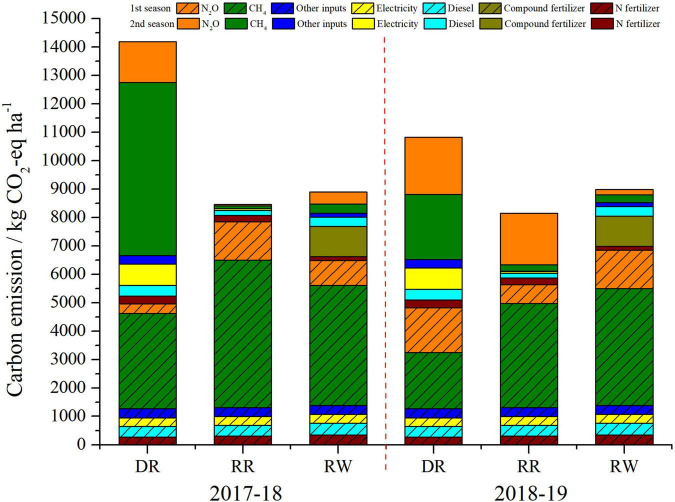
Average contribution of different sources to the carbon emission of the three cropping systems. DR, double-rice system; RR, ratoon rice system; and RW, rice-wheat system.

The average contribution of different sources to the carbon emissions is illustrated in Figure 3. It is clear that methane on-field emission was the largest cause of the whole carbon emissions with a percentage of 39.38–66.48%, closely followed by the on-field nitrous oxide, the second important source, accounting for 12.61–33.21%. Indirect GHG emissions from agricultural inputs together accounted for 20.91–34.30%, in which, synthetic fertilizer, diesel for mechanical cultivation, and electricity for irrigation were the most important sources to the carbon emissions (Table 5 and Figure 3). Furthermore, compound fertilizer in RW system was the primary single agricultural input source accounting for 11.82–11.93% of the total GHG emissions.

**TABLE 5:** Yearly average greenhouse gases (GHGs) and reactive nitrogen (Nr) emissions for the three rice cropping systems.

Items	GHG emission (kg CO_2_-eq/ha)			Nr emission (kg N-eq/ha)		
	DR	RR	RW	DR	RR	RW
**Indirect emissions**						
N fertilizer	550.8	535.5	481.95	0.32	0.312	0.28
P fertilizer	244.5	122.25	122.25	0.081	0.041	0.041
K fertilizer	214.5	117	117	0.01	0.005	0.005
Compound fertilizer	0	0	1062	0	0	0.138
Diesel	748.5	540.57	748.5	0.804	0.581	0.804
Electricity	1049.68	380.19	307.5	0.154	0.056	0.045
Herbicides	43.52	21.76	21.76	0.009	0.005	0.005
Insecticides	16.24	8.12	8.12	0.007	0.004	0.004
Fungicides	4.83	2.42	4.52	0.003	0.002	0.003
Labor	25.8	17.2	14.62	0	0	0
Rice seed	69	34.5	34.5	0.029	0.014	0.014
Wheat seed	0	0	130.5	0	0	0.054
**Direct emissions**						
CH_4_	6,849.67	4,583.67	4,476.5			
N_2_O	2,692.43	1,940.97	1,413.51	9.04	6.51	4.74
NH_3_				51.71	50.27	59.03
NO_3_^–^				25.04	24.35	25.99
NH_4_^+^				1.23	0.58	0.79
Total	12,509.47	8,304.15	8,943.23	88.44	82.73	91.94

*DR, double-rice system; RR, ratoon rice system; RW, rice-wheat system.*

Table 4 shows the CF performance of the three cropping systems. The total CF were from 483.98 to 1,094.13 kg CO_2_-eq t^–1^ among the three modes during the 2 years. In 2017–2018, DR system had the highest CF at 1,094.13 kg CO_2_-eq t^–1^, significantly higher than those of 604.99 kg CO_2_-eq t^–1^ for RW and 567.22 kg CO_2_-eq t^–1^ for RR (*P* < 0.05), but no significant difference between RW and RR. In 2018–2019, DR maintained the highest CF of 718.97 kg CO_2_-eq t^–1^, 24.27 and 48.55% higher than RW and RR (*P* < 0.05), respectively. In addition, RW was 19.54% higher than RR. The interaction between cropping systems and year had a highly significant effect on CF (*P* < 0.01). Specifically, the CF of the three systems in 2 years presented the following order: DR > RW > RR.

### Nitrogen Emissions and Nitrogen Footprint

**FIGURE 4:**
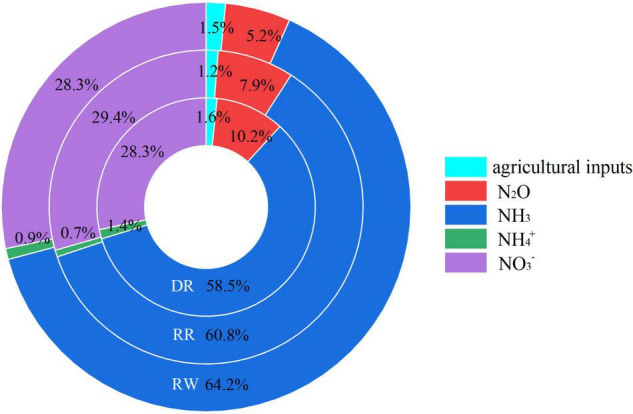
Contribution of different sources to the reactive N emission of the three cropping systems. DR, double-rice system; RR, ratoon rice system; and RW, rice-wheat system.

Table 5 and Figure 4 show that NH_3_ volatilization was the principal source to the Nr emissions with the proportion of 58.47% to DR, 60.76% to RR, and 64.20% to RW, respectively. The second significant contributor to the Nr emissions was NO_3_^–^ leaching, which accounted for 28.27–29.43% among the three rice cropping systems. Additionally, N_2_O had a percentage of 5.16–10.22% to the N emissions as the third important source. In contrast, all of the agricultural inputs and NH_4_^+^ had little contribution to the total nitrogen emissions, accounting for only 1.93–3.00% taken together.

Ratoon rice system showed the lowest NF of 5.41 and 5.03 kg N-eq t^–1^ in 2017–2018 and 2018–2019, respectively (Table 4), significantly lower than those of DR and RW in both years (*P* < 0.05), while the highest NF value was observed in DR, which were 6.59 and 6.07 kg N-eq t^–1^ in the 2 years, respectively. Obviously, the NF value of RW was somewhere between DR and RR. Furthermore, no significant differences existed between RW and DR.

### Water Footprint Performance

The WF value for DR system was found to be the highest at 1,338.33 and 1,103.79 m^3^ t^–1^ in the two study years, respectively (Table 4), 24.52% higher than RR and 13.06% higher than RW in the first year (*P* < 0.05), while the percentages were 21.40 and 9.56% in the second year. WF values for the RW system were 10.13 and 10.81% higher than those of the RR within 2 years (*P* < 0.05).

**FIGURE 5:**
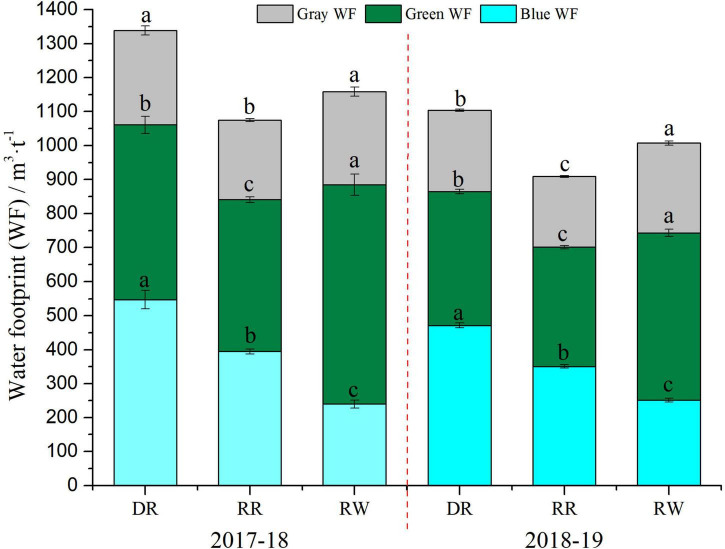
Water footprint for the three rice cropping systems. DR, double-rice system; RR, ratoon rice system; RW, rice-wheat system. Different lower-case letters indicate the significant differences (*P* < 0.05) in a same individual water footprint.

Figure 5 shows that green, blue, and gray water contributed 35.64–38.37, 40.90–42.71, and 20.74–21.66%, respectively, to the whole WF for DR, while the proportions of the three categories of water for the RR mode were respectively 38.55–41.47, 36.73–38.57, and 21.79–22.88%. In addition, green water (48.85–55.72%) was the largest contributor to WF for RW. Noticeably, the blue WF among the three rice cropping systems demonstrated the order of DR > RR > RW in both years, which showed the difference in irrigation water use. Additionally, the RR mode had the smallest WF value, significantly lower than the other two modes.

## Discussion

### Effects of Different Cropping Systems on Carbon Footprint

In this study, the CF values among the three rice cropping systems over the 2 years were 483.98–1,094.13 kg CO_2_-eq t^–1^ (Table 4), which were consistent with many previous studies in central or southern China (Liu et al., 2016; Xue et al., 2016; Chen et al., 2020; Xu et al., 2020a). However, Jiang et al. (2020) reported a slightly higher CF value of DR pattern since they adopted higher direct and indirect emission factors. Liu et al. (2020d) reported a higher CF in southern China mainly because of more intensive machinery operation and extensive use of plastic films. A significantly higher CF in a DR system reported by Sun et al. (2019) possibly attributed to the adoption of higher emission factors of agricultural inputs. Results from this study are higher than the research by Cheng et al. (2015), since their calculation of CH_4_ and N_2_O were based on lower statistical data and emission coefficients.

In this research, direct GHG emissions, especially methane on-field emission, constituted the largest fraction to CF in all of the three rice cropping systems (Figure 3), which was consistent with many previous studies (Xue et al., 2016; Jiang et al., 2020; Xu et al., 2022). Therefore, reduction of field methane emissions would be an effective strategy for reducing CF; there are a number of appropriate farming practices that can serve this purpose. For example, Fertitta-Roberts et al. (2019) reported that noncontinuous flooding can reduce methane emissions by 44%; Liu et al. (2020d) found that N fertilizer deep placement can reduce methane on-field emission by 36–39%; Jiang et al. (2019) reported that optimizing the amount of N fertilizer can reduce GHG emissions. Additionally, synthetic fertilizer was the principal component of GHG emission from agricultural inputs (Table 5 and Figure 3), similar with the results reported by Chen et al. (2020) and Xue et al. (2016); in addition, indirect GHG emission from electricity accounts for up to 35.37% in DR system mainly because DR system needs a lot of electric power for pumping irrigation. Yu et al. (2021) suggested that GWP of the second season was significantly lower than the first season in RR, which is similar to our result. Furthermore, Figure 3 shows that CH_4_ emissions of the three cropping systems in 2017–2018 were all higher than that of 2018–2019, when it came to N_2_O; the results were opposite. This phenomenon could be explained by the following reasons. Methane is only produced rely on the decomposition of soil microorganisms under anaerobic conditions (Fagodiya et al., 2017), while nitrous oxide is primarily produced through nitrification and denitrification under aerobic conditions (Bouwman, 1998). However, rainfall during the rice growing season in 2018 was 7 days less than usual, and the temperature was 1.4° higher on average (Hubei Meteorological Service [HMS], 2018). Under the condition of sufficient oxygen, high temperature, and dry soils in 2018, N_2_O emissions would be enhanced while the production of CH_4_ was severely inhibited.

In this study, cropping systems have significant effects on yield and CF (*P* < 0.01) and the general trend of CF in the two study years being DR > RW > RR (Table 4). Similarly, Chen et al. (2020) showed that the CF for DR was 12.17% higher than RW, mainly attributed to higher CH_4_ field emission of DR. Xu et al. (2022) indicated that the RR system reduced average annual CF by 27.37% compared with the DR system, which may be due to the annual CF of the second season in RR was significantly lower than that of DR. Therefore, it demonstrated the possibility of rational selection of cultivation mode to balance yield and footprint impact, which is consistent with many previous studies (Ali et al., 2012; Cha-un et al., 2017; Sun et al., 2019).

### Effects of Different Cropping Systems on Nitrogen Footprint

In this study, the NF value for the three systems was 5.03–6.59 kg N-eq t^–1^ across the two test years (Table 4), which were higher than the NF of a rice monoculture system in central China (Xu et al., 2020a) mainly because of the lower application of the N fertilizer rate. Chen et al. (2020) and Xue et al. (2016) reported higher average NF for DR and RW in southern China since they employed higher NH_3_ and N_2_O emission factors; Pierer et al. (2014) reported a significantly higher NF of cereals in Austria, possibly because they calculated the NF of not only the production process but also the consumption process. In addition, NH_3_ volatilization was the primary source of active N emission among the compared cropping patterns (Table 5 and Figure 4), which agrees with previous studies (Leip et al., 2014; Chen et al., 2020; Xu et al., 2020b). Therefore, the selection of NH_3_ volatilization coefficient was very important for the calculation of NF; thus, we chose a NH_3_ volatilization coefficient obtained from the long-term location tests in the study site, which was close to other test results in central China (Qi et al., 2012; Li et al., 2018). Moreover, Chen et al. (2020) and Xu et al. (2020b) suggested NO_3_^–^ was the second major source to the NF, which was consistent with our result. A large number of previous studies showed that Nr was closely related to the N fertilizer applications (Qi et al., 2012; Pierer et al., 2014; Xu et al., 2020b). So proper nitrogen fertilizer management is crucial to the mitigation of Nr without compromising the grain yield, such as reasonably adjust the amount of nitrogen application (Qi et al., 2012) or selecting appropriate fertilizer categories (Lian et al., 2018). It is difficult to compare the NF value with others owing to the limited research on NF of the three rice cropping systems. Similar to the CF, cropping systems have a significant effect on NF (*P* < 0.01), and the NF value follows the tendency of DR > RW > RR (Table 4). On the contrary, Chen et al. (2020) found that NF of DR was lower than RW by 13.43%, primarily because RW had a lower grain yield.

### Effects of Different Cropping Systems on Water Footprint

The WF of the three rice cropping systems in this study ranged from 909.21 to 1,338.33 m^3^ t^–1^ (Table 4), which were similar to some studies (Xu et al., 2020b; Zhai et al., 2021). Chapagain and Hoekstra (2011) also reported the green, blue, gray, and total WF of rice production in China, respectively, were 367, 487, 117, and 971 m^3^ t^–1^, while the values of global average WF of rice planting were 346, 374, 65, and 784 m^3^ t^–1^. The difference mainly came from gray WF due to the higher N fertilizer rates. Kashyap and Agarwal (2021) indicated higher WF value of rice and wheat in India, primarily due to the higher blue WF; local farmers used a lot of irrigation water under drought conditions. In addition, the average blue WF followed the order: DR > RR > RW (Figure 5) possibly because DR still needs a lot of irrigation water in the second season, which is significantly higher than that of RR, while RW only needs natural precipitation instead of irrigation water in the second season, which is also the reason why RW has the highest proportion of green WF. Moreover, due to the different nitrogen application rates and yield factors, both RW and DR had significantly higher gray WF than RR (Figure 5). Therefore, the key to reducing WF lies in water-saving irrigation and nitrogen reduction (Zhang et al., 2014; Livsey et al., 2019).

In this study, year and cropping system had profound impacts on grain yield and WF (Table 4). In fact, the total amount of virtual water in 2017–2018 was only 4.43–11.17% higher than that in 2018–2019, but the WF of the former was 17.50–21.25% higher than that of the latter, which was mainly because grain yield of all treatments in 2018–2019 were higher than that in 2017–2018 due to a better meteorological conditions (Figure 1).

### Policy Suggestion for System Selection

Grain yield and environmental impacts need to be considered during rice production decision-making (Zhu et al., 2016). Compared with DR and RW systems, RR was a low-cost and high-output cultivation mode not only because of the low labor intensity but also because of the feature of harvesting twice in a single year while only sowing once (Tables 2, 3). Our results also illustrated that the RR system had a lower environmental impact due to its performances on CF, NF, and WF, but with the highest grain yield (Table 4), which was consistent with a research (Xu et al., 2022). The RR system has become a significant cropping system promoted over a wide region of southern China since the 1980s. Meanwhile, there are 3.3 million ha rice fields suitable for growing ratoon rice accounting for 30.8% of the total paddy fields in southern China (Xu et al., 2022).

Furthermore, it is important to take into account the current social environment and rural situation when appropriate agricultural policies are established (Pandey and Agrawal, 2014; Zhou et al., 2022). First, the population issue cannot be ignored because the rural population accounts for only 36.11% of the total population with a sharp decrease by 164.36 million during 2010–2020. Second, the population older than 60 years accounted for 18.70% compared with 13.26% in 2010; therefore, the problem of an aging population will deepen (NBS, 2022). Finally, the interaction of rural land transfer and urbanization also has an impact on the rural population (National Bureau of Statistics [NBS], 2021). All of these factors have resulted in a large reduction in the rural laborers; therefore, the low labor intensity rice planting mode (i.e., RR) has become an important choice.

## Conclusion

Considering the future conditions of rural societal developments and rapid demographic changes, our results highlight that the RR system could be an important tradeoff of grain yield and environmental impacts. Additionally, reasonable nitrogen application and irrigation are helpful to reduce the impacts of CF, NF, and WF and maintain stable yield. Although the 2 years of experiment was conducted in the typical rice cultivation region in central China, there were still many uncertain factors in the assessment of CF, NF, and WF at a regional scale due to spatial and soil heterogeneity. Therefore, long-term and regional-scale experiments are needed to obtain comprehensive detailed information on CF, NF, and WF.

## Data Availability Statement

The original contributions presented in the study are included in the article/supplementary material, further inquiries can be directed to the corresponding authors.

## Author Contributions

YZ, BZ, and ZL initiated and designed the research. YZ and SG performed the experiments. KL, MH, SF, BZ, and ZL revised and edited the manuscript and provided advice on the experiments. All authors contributed to the article and approved the submitted version.

## Conflict of Interest

The authors declare that the research was conducted in the absence of any commercial or financial relationships that could be construed as a potential conflict of interest.

## Publisher’s Note

All claims expressed in this article are solely those of the authors and do not necessarily represent those of their affiliated organizations, or those of the publisher, the editors and the reviewers. Any product that may be evaluated in this article, or claim that may be made by its manufacturer, is not guaranteed or endorsed by the publisher.
